# A Comparison of Constructions of Spirituality in an Australian Nursing Context

**DOI:** 10.1177/08980101251336323

**Published:** 2025-05-04

**Authors:** Katherine Louise Cooper, Kathleen Dixon, Esther Chang, Lauretta Luck

**Affiliations:** School of Nursing and Midwifery, Western Sydney University, Parramatta, New South Wales, Australia; School of Nursing and Midwifery, Western Sydney University, Parramatta, New South Wales, Australia; School of Nursing and Midwifery, Western Sydney University, Parramatta, New South Wales, Australia; Centre Nursing and Midwifery Research, School of Nursing and Midwifery, Western Sydney University, Parramatta, New South Wales, Australia

**Keywords:** spiritual, holistic, religious beliefs, empathetic, professionalization

## Abstract

**Purpose of study:** The purpose of this study was to explore whether the professional construction of spirituality resonates with the experience and understanding of registered nurses (RNs). **Design of study:** Fairclough's critical discourse analysis approach. **Methods used:** Participants comprised three RN Standards for Practice research and development team members and 20 RNs working in hospitals in Australia. Data analysis was conducted through the application of Schneider's ten work steps. **Findings:** Holistic discourse was found to be a common discourse among both groups of participants. Other discourses which the two groups of participants drew from in their constructions of spirituality were mostly unique to each group. **Conclusions:** The findings have implications for spiritual care practice in the context of holistic nursing, notably in nursing practice standards and nursing education.

## Introduction

Spirituality in the context of nursing has been explored in recent decades (Domingo-Olse, 2020; Lalani, 2020). However, it is apparent some confusion exists regarding the meaning of spirituality (Cooper et al., 2019; Egan et al., 2017; Lalani, 2020; Murgia et al., 2020) with nurses finding it difficult to interpret and action spirituality in practice (Lalani, 2020). Spirituality as a concept originates from a Latin word “spiritus,” which refers to breath, soul, character, or inspiration (Chiu et al., 2004) and can be part of an individual's worldview and value system (McSherry & Jamieson, 2011). Various meanings have been ascribed to spirituality ranging from existential meanings such as purpose and meaning in life (Cooper et al., 2019; McSherry & Jamieson, 2013) to religious perspectives (Chiang et al., 2020; Melhem et al., 2016). Spirituality is also viewed as a well-being and quality-of-life concept which forms part of holistic care (Blasdell, 2015). Holistic is the adjective of the theoretical concept of holism, which is based on the understanding the individual is a whole person consisting of an inseparable integration of body, mind, and spirit (Narayanasamy et al., 2004; Tjale & Bruce, 2007).

Some nursing authors have criticized definitions of spirituality used in nursing. They argue despite the fact nurses have written extensively about the positive characteristics of spirituality, there has been very little critique of how spirituality is defined in the nursing context (Draper & McSherry, 2002; Henery, 2003; Swinton, 2006; Swinton & Narayanasamy, 2002). Clarke (2009) proposes the deficiency of critique has led to spirituality being defined through broad, existential definitions which separate spirituality's relationship to religion or theology. Thus, the concept of spirituality is left open to be confused as only relating to psychosocial constructs (Reinhart & Koenig, 2013). The multitude of spirituality definitions has been blamed for making spirituality a relatively meaningless concept able to be covered in psychology or other aspects of nursing curricula (Paley, 2008a, 2008b; Swinton & Pattison, 2010).

The lack of concise definition of spirituality has possibly led to the lack of professional guidelines for nurses in ways to provide spiritual care (Ross, 1994) and nurses not knowing how to provide spiritual care (Ross, 2006). In Australia, the RN Standards for Practice are used to guide the clinical practice of RNs. The purpose of these standards is to describe nursing practice for government regulation, employers, education providers, and consumers and is important to ensure quality care provision (Cashin et al., 2017). The current Australian RN Standards for Practice does not contain the word spirituality but contain the word “holistic.” With spirituality commonly recognized as forming part of holistic care, there is a need to consider spirituality in relation to the nursing standards for practice and how spirituality is understood by registered nurses (RNs) who are expected to provide holistic care in practice. It is unknown whether those involved in developing the currently used RN Standards for Practice's constructions of spirituality within holistic care align with those of the RNs using these standards. The aim of this paper is to report on a study which sought to find out whether the professional construction of spirituality resonates with the experience and understanding of RNs.

## Methods

### Aim

To compare the discursive constructions of spirituality of members of the RN Standards for Practice research and development team and RNs working at two Australian hospitals.

### Design

This study used Fairclough's critical discourse analysis approach for analyzing and interpreting textual data. Using Fairclough's critical discourse analysis approach exposed hidden assumptions and questioned the status quo regarding spirituality and spiritual care in the context of nursing by revealing how power has shaped these assumptions. Critical discourse analysis is a methodological approach centers on understanding discourse, an act of communication through the use of language (Johnstone, 2018; Strauss & Feiz, 2014). Combining the word critical with discourse analysis acknowledges the connection of social practice with language (Strauss & Feiz, 2014). Fairclough's (Fairclough, 1995) approach to critical discourse analysis conveys an understanding of the connection between discourse and power (influences) and focuses on how language is used. Fairclough (Fairclough, 2003) based his approach on the supposition that language forms a complex part of social life which is linked to other constituents of social practice. In relation to this study, social practice refers to nursing practice. The interview texts were critically appraised for language used regarding spirituality and the power mechanisms influencing participants’ constructions of spirituality. The use of language was critical to how the participants constructed spirituality and practiced spiritual care.

### Participants

This study comprised of semistructured interviews with two participant groups selected to participate in the study using a purposive sampling method (see Table 1 and Table 2). The first group of participants consisted of three members of the RN Standards for Practice research and development team. The team comprised of ten members but only four could be accessed and three agreed to be interviewed. These participants were integral to the developing the standards and were in a good position to talk about the representation of spirituality in the standards (Cashin et al., 2017). The second group of participants comprised of 20 RNs: 14 RNs from a faith-based private hospital and six RNs from a nondenominational public hospital in Sydney, Australia. These settings were chosen because the RN participants working within these hospitals were from varied cultural, religious, and educational backgrounds which were seen to be important for gaining a wider understanding of how RNs construct spirituality. The participants worked in a range of clinical areas, including medical, surgical, oncology, and critical care.

The RN Standards for Practice research and development team participants were questioned about their perspectives on how spirituality is represented in the RN Standards for Practice and influences affecting its representation. The RN participants were asked about the meanings they ascribe to spirituality and spiritual care, and what influences affected the meanings they ascribed to these terms.

### Recruitment and Data Collection

Following ethical approval from the university and hospitals’ ethics committees, both groups of participants were approached to invite them to participate in the study. The RN Standards for Practice research and development team were contacted via email following identification of the members of the team from publically available documents such as the Cashin et al. (2017) article, whereas the RNs were approached following permission from their Nursing Unit Managers. Each potential participant received a participant information sheet which contained a written explanation of the aims of the study and what participation involved. For those interested in participating, dates and times for interviews were agreed upon. The interviews with the RN Standards for Practice research and development team took place via telephone and internet (zoom) technology due to the diversity of their locations, whereas the interviews with the RNs were conducted face to face then via telephone during the coronavirus (COVID-19) restrictions. Data for both groups of participants were collected through the use of in-depth semistructured interviews. The interviews were recorded and transcribed.

### Ethical Considerations

This study was conducted according to the principles of ethical conduct outlined in the Australian National Statement of Ethical Conduct (2018). The ethics committees of both hospitals and the university ethics committee granted approval for this study. Before participating in this study, all participants gave their informed consent voluntarily. To maintain confidentiality, participants were given pseudonyms.

### Data Analysis

Schneider's (Schneider, 2013) ten work steps were used to analyze the interview texts of both groups of participants. Schneider's work steps were underpinned by Fairclough's (Fairclough, 1995) critical discourse analysis approach. Schneider also drew from Chilton's (Chilton, 2004) understanding of a link between politics and language and Jager's (Jäger, 2004) discourse analysis steps. Schneider's work steps provided a distinct framework for applying Fairclough's critical discourse analysis method to data analysis.

Firstly, in alignment with Schneider's initial work steps, the historical and social context of the text source (participant interviews) were examined to reveal any underlying power relations which may have influenced the participants’ expressions of spirituality. This included the participants’ present workplace and educational background. Doing this enabled a greater insight into how these discursive contexts shaped the participants’ understandings of spirituality and spiritual care.

Next, the data were entered onto a computer and coded according to keywords and themes and placed into a table, in accordance with Schneider's (Schneider, 2013) work steps 3 and 4. Following this, in alignment with Schneider's work steps 5–8, the data were examined for discursive statements, structural features, and linguistic methods such as grammar and modalities used within the text. In relation to this study, these steps were applied to uncover mechanisms of power and assumptions which may have influenced expressions about spiritual care and spirituality from the participants. Finally, the findings were used to answer the research question. This was in alignment with Schneider's final two work steps 9 and 10.

### Rigor

To ensure rigor in the study, attention was paid to the study's credibility, trustworthiness, and dependability in the following ways. The principal researcher's supervisory panel presided over the conduct of the research and interpretation of findings. The supervisors’ experiences in conducting qualitative and critical discourse analysis research helped in attaining credible findings. The researcher's supervisory panel examined all transcriptions, documentation of the data analysis steps, findings, and discussion. This was done to discover claims or biases not verified by the data to make sure the findings authentically reflected the participants’ experiences (Whittemore et al., 2016). This process assisted in preventing the principal researcher's views of spirituality's importance from influencing interpretation of the findings. Exemplars were drawn from the interview transcriptions to support the findings (Polit et al., 2018).

### Findings

The discourses of spirituality of both groups of participants are listed in Table 3. These discourses were compared to find out whether the professional construction of spirituality resonates with the experience and understanding of RNs.

**Table 1.** Participant Demographics: RN Standards for Practice Research and Development Team

Gender	Male	2
	Female	1
Professional background	Nursing	2
	Non-nursing	1

**Table 2.** Participant Demographics: Registered Nurses from Faith-Based Private and Nondenominational Public Hospital

Gender	Male	2 (10%)
	Female	18 (90%)
Age	21–30	11 (55%)
	31–40	4 (20%)
	41–50	1 (5%)
	51–60	4 (20%)
Years of experience	<5	12 (60%)
	6–10	3 (15%)
	11–20	2 (10%)
	>20	3 (15%)

**Table 3.** Participant Group and Spirituality Discourses

Participant group	Discourses
Members of the RN Standards for Practice research and development team	Spirituality part of holistic care Spirituality part of person-centered care Professionalization of nursing
RNs from a faith-based private hospital and nondenominational public hospital	Spirituality as part of holistic care Spirituality as personal religious beliefs Spirituality as empathetic care

### A Shared Discourse

#### Spirituality Part of Holistic Care

The findings suggest there was one discourse in common with members of the RN Standards for Practice research and development team participants and the RN participants, as highlighted in Table 3. This was the discourse of holistic (adjective form of holism) care which featured in the interviews of participants from both groups. Almost all participants located their understanding of spirituality within holistic discourse with most participants including spirituality as a component of holistic care. This is illustrated in the following text extracts:

Spiritual care is one element of holistic care and it was just about trying to embed the notion of holistic care, to see the person in all of their dimensions. (Janelle, standards developer)

Holistic is meeting the person's all round needs, all their spiritual beliefs, their mental, and physical needs… just everything the person needs in order to feel they're living a fulfilled life. (Elizabeth, RN)

Holistic means the spiritual aspect as well as all the other aspects of care. I just think today, this world, it's a bit disappointing, but I think people are on the fast train and moving so quickly through life that the time of reflection, the time of being in touch with his spiritual side is sometimes neglected, until things cause them to stop. (Sonia, RN)

Although both groups resonated with constructing spirituality through holistic discourse, the influences for constructing spirituality through holistic discourse were different for each group. Resonance with the term holistic was attributed by the members of the RN Standards for Practice research and development team participants to the general acceptance of holism in nursing and health. This is exemplified in the following text excerpts.

The nuancing has moved, and holism it's become accepted, people are more than a group of physical symptoms. That health is more than the absence of disease, and new terms in the old standards, where they had to be spelled out, now are taken for granted assumptions. (Mark, standards developer)

The vision that nursing is holistic and it's focused on treating symptoms of ill health. (Janelle, standards developer)

The inclusive nature and holistic perspective we're trying to promote here in the standards to encompass the person in their world among their loved ones who has their own values and spiritual beliefs and attitudes. (Karl, standards developer)

Whereas the RN participants mostly attributed their understanding of spirituality to what they had learned about holistic care during their undergraduate education, thus showing the power of preregistration education on shaping RNs’ construction of spirituality. This is illustrated in the following text extracts:

I was taught to treat the patient holistically, physically, mentally, spiritually, and if somebody is good mentally and spiritually, they will get better physically as well. (Mary, RN)

I think uni pointed out quite well, it's holistic care, so it's not just giving pills and taking a blood pressure, it's meeting their entire needs, including spiritual needs. (Gillian, RN)

The other discourses which emerged in both groups’ constructions of spirituality were dissimilar and unique to each group of participants. The discourses of person-centered care and professionalization of nursing emerged for the RN Standards for Practice research and development team participants. Whereas for the RN participants, the discourses of personal religious beliefs and empathetic care were revealed.

### RN Standards for Practice Research and Development Team Discourses

#### Spirituality Part of Person-Centered Care

In addition to holistic discourse, all the members of the RN Standards for Practice research and development team participants also constructed spirituality through person-centered care discourse. The participants understood person-centered care, like holistic care, to be an encompassing term that included spirituality. The discourse of person-centered care was drawn from trends toward person-centered care in nursing and health. This is illustrated in the following text excerpts:

There is the much more full emergence of the person-centered care, and what that means, not just as a rhetoric but how nurses need to live that. (Mark, standards developer)

More about the trend genuinely toward person-centered care. (Janelle, standards developer)

### The Professionalization of Nursing

The members of the RN Standards for Practice research and development team participants recognized the importance of challenging stereotypes through representing nursing as a profession. The interview texts of these participants revealed widely held historical stereotypes of nurses and nursing influenced the writing of the standards. Mark expressed there are “*impoverished views of nursing*” by those outside of nursing. Karl conveyed how the standards needed to express the capabilities of nurses as professionals. This is explicated in the following interview text:

I was very aware of the history of how politicians have often viewed nursing, and I'm very aware of the reductionist view that's often expressed politically, or sometimes sociopolitically on nurses. That's our historical perception on nursing, which certainly very much influenced the standards. Therefore, the standards have to make sure they're presented as the capabilities of the professional, and this is what the public will expect of this professional. (Karl, standards developer)

The participants saw the development of specific and measurable professional standards which can readily be applied to all nurses working in all contexts of practice in Australia as a means of upholding nursing as a profession. With spirituality considered a term not easily measured, it was not included in the standards. This is elucidated in the following extracts from Karl's (standards developer) interview text:

It (spiritual) is a very hard one to measure, let alone write specifically

Generally, with a standard, it's an outcome. It advocates on behalf in a manner which respects a person's autonomy. You're looking at specific and measurable outcomes. Whereas underpinning how you do that relates to spiritual.

In summary, both groups of participants constructed spirituality through holistic discourse. The standards developers also constructed spirituality through person-centered care and professionalization of nursing discourses. The next section will discuss RN spirituality discourses.

### Registered Nurse Discourses

#### Spirituality as Personal Religious Beliefs

On the other hand, the responses of the RN participants from both hospitals suggested their constructions of spirituality were largely personalized. In particular, the personal religious beliefs of the participants were found to shape the participants’ spirituality constructions. Almost all interview texts showed evidence of interpreting spirituality through their personal religious beliefs. The following text extracts illustrate this:

Because for me, spirituality was more about your religious belief, and how you function. How you allow the Holy Spirit to direct your life. (Elizabeth, RN)

Spirituality, my definition I think it's how do you say it, a higher power. Some people would have God, some people would have Buddha, which I respect. (Peter, RN)

Some participants who did not consider themselves religious drew from their family member's religious perspectives as exemplified in Jemima's statement below.

I've had religious grandparents and I understand some people really do believe in heaven. For spiritual care, she did the prayer, but we both said “Amen” at the end. (Jemima, RN)

This finding indicates personal religious beliefs and family members’ beliefs had a powerful influence on how the participants constructed spirituality and practiced spiritual care.

### Spirituality as Empathetic Care

In addition to this the findings showed the RN participants described their understanding of spirituality through empathetic care constructs. These empathetic perspectives were expressed in a way which indicated deep personalization of spirituality. This is illuminated in the following interview texts:

Everyone has a certain spirituality which can even be an energy it can be translated into our practice by just being more of an empath to a patient than just a clinical. (Jane, RN)

You might get some patients which are quite demanding, you might get some patients because they're lovely but then you get some patients that are confused. Spirituality is listening to what they want, being empathetic. (Sara, RN)

Spirituality… so, putting myself in their position. Like I see old patients like old people get frustrated and they yell at you, but I understand them, I don't mind because I know I'll be like that. (Hannah, RN)

This text extracts suggest the participants’ personal empathetic engagement with patients was viewed as a means of providing spiritual care.

### Influence of Education

The findings suggest the discursive practices (what the nurses practice in their care informed by discourse) of the participants in relation to spiritual care were reinforced by education about spirituality in their preregistration nursing program and the ideology of the hospital they were working in at the time of the interviews. Most of the participants made references to the way spirituality was taught in their undergraduate programs and the impact it had on their understanding of spirituality and practice of spiritual care. This is illustrated in the following statements:

Learning those spiritual units back in uni its helps a lot, gives us a lot of understanding while we are providing care and making decisions. I think in that area its really gives a lot of influence on how we think for the patients from their different aspect not from a medical, from a spiritual. (Isla, RN)

So I think university really helped shape that (spiritual care) for me, and just sort of implementing ways I can do that in providing patient care. I'd say probably had a big significant impact on the spirituality side. I do think it's a really important part of nursing. (Angela, RN)

Thus educational background served as a discursive construct as it exerted power on the RN participant's understanding of spirituality, particularly those who had completed their undergraduate nursing programs at private religious institutions and were presently working at the faith-based private hospital.

The ideology of the hospitals as exemplified in their mission statements also exerted an influence on the RN's understanding and practice of spirituality and spiritual care. The faith based private hospital's mission statement centers on the religious connection of the hospital. The responses of the participants working within this hospital indicated they resonated with the mission statement when relating their understandings of spirituality from a personal religious beliefs and/or holistic perspectives of care. This is exemplified in the following statements:

The hospital has a vision and mission I can connect with my spiritual care practice and understanding. (Gillian, RN)

I think when people come to a religious institution. They have different expectations. You kind of feel like this is a safe place to express your spirituality because of what the institution represents, the key mission statement of this hospital. (Mary, RN)

The nondenominational public hospital participants' responses likewise showed their alignment with the hospital's values, visions, and goals such as openness and respect when discussing their perspectives on spiritual care and spirituality. The responses indicated a personalization of these perspectives as evidenced in the following statements:

I think I am doing the right thing, spirituality for myself but then I respect everybody else, those who will come through the door of our ward. It should be recognized and it should be respected. (Louise, RN)

Because people will draw on their own personal spiritual practices in times of distress and illness. And we have to be open to allowing that and not imposing our own spiritual practices. (Victoria, RN)

## Discussion

The findings are now discussed in relation to mechanisms of power and assumptions which may have influenced expressions about spirituality and spiritual care. Only one discourse, holistic care, was common with both groups of participants. Otherwise spirituality was mostly constructed in dissimilar ways unique to each group. The difference in discourses among both groups of participants is likely attributed to the impact of different power mechanisms.

The RN Standards for Practice research and development team were in a powerful position to develop the standards. They were invested with power from the most influential nursing organization in Australia, the Nursing and Midwifery Board, Australia. Although a large number of stakeholders were engaged in the process of the development of the standards (Cashin et al., 2017), the ten members of the RN Standards for Practice research and development team had the power to decide what was included and excluded from the standards. The findings of this study indicate challenging historical stereotypes of nurses and nursing exerted an influence on the RN Standards for Practice research and development team participants’ constructions of spirituality and how they viewed spirituality's absence from the standards. Furthermore, the social power of present trends in nursing practice of holistic and person-centered care (Chinn & Kramer, 2018; Mandal et al., 2019; Puchalski, 2013; Vincensi, 2019) influenced these participants’ constructions of spirituality in spiritual care was viewed as embedded within understandings of holistic and person-centered care.

In contrast, the findings of this study indicate the RN participants’ constructions of spirituality were highly personalized and significantly influenced by their own individual perspectives as well as their educational background and work environment. For the RNs who held religious beliefs, their religious background exerted power over their constructions of spirituality and spiritual care. Other studies have also found nurses relating spirituality to religion (Chew et al., 2016; McSherry & Jamieson, 2013; Melhem et al., 2016), which could be attributed to the power of the nurse's religious beliefs on their constructions of spirituality. For some participants in this study, their family background also had an influence.

Unlike the RN Standards for Practice research and development team participants, professionalization of nursing was not mentioned by the RN participants. They focused more on issues relating to doing the right thing by their patients and themselves in their day-to-day practice. The social power of preregistration education, where the RN participants had been taught about spirituality and spiritual care, especially as spirituality relates to holistic and religious perspectives, influenced their constructions of spirituality. This finding is consistent with other studies which have found education in preregistration nursing programs has had a positive impact on preparedness of RNs and students in nursing to provide spiritual care in practice (Cooper & Chang, 2016; Melhem et al., 2016; Ozbasaran et al., 2011; Wu et al., 2012; Wu & Lin, 2011). Moreover, for those participants who were working in the faith-based private hospital, the cultural power of the institutional environment reinforced their constructions of spirituality. Melhem et al. (2016) also found institutional environment had an impact on RNs. With RNs working in private institutions having higher spirituality and spiritual care perceptions than those working in government institutions.

The findings highlight not only the personalization of spirituality for RNs in practice but the dissonance in constructions of spirituality and spiritual care between those setting the standards and those in practice. The findings also show the complexity of spirituality as a contested term with a range of meanings and the associated challenges in trying to capture a unified understanding of what spirituality means. Incorporating a contested term such as spirituality in nursing policies, practice, and education presents a challenge. The following section will discuss the implications of the findings on policy, practice, and education.

### Implications for Education, Practice, and Policy

The findings of this study have implications for holistic nursing practice. This study revealed practicing spiritual care is highly personalized being significantly influenced by the religious beliefs of the RN. With Chiang et al.'s (Chiang et al., 2020) study showing the religious beliefs of nurses positively impacting their perspective and practice of spiritual care, this could be seen as a positive finding. However, the high degree of personalization of spirituality among the RN participants, especially in relation to the RNs’ religious beliefs also poses a risk to the provision of spiritual care. Personalizing spirituality through the religious beliefs of the RN could lead the nurse to provide holistic, especially spiritual care based on their own beliefs (Fawcett & Noble, 2004). Providing care based on the nurses’ religious beliefs could be problematic for a patient with a different religious or nonreligious background, with some patients possibly uncomfortable with receiving such care. Asking the patient what is meaningful to them could be an appropriate holistic and person-centered approach to providing spiritual care based on the patient's preferences.

Regarding the education of RNs, this study discovered a lack of a shared understanding about spirituality among participants from both groups with only the discourse of holistic care being common. With spirituality discourses being largely dissimilar between both groups, this poses a challenge for education providers incorporating spirituality teachings within their programs. The lack of coherent understanding of spirituality could impact the incorporation of spirituality into the education of RNs which coupled with spirituality's absence in the RN Standards for Practice may account for many education providers not educating RNs about spiritual care as a part of holistic care (Southard, 2020; Vincensi, 2019). However, not educating RNs about spiritual care can leave them unprepared to address the spiritual needs of their patients, leaving holistic care provision incomplete (Southard, 2020) and not fully compliant with the AHNA Scopes and Standards of Practice (AHNA, 2019). The AHNA Scopes and Standards of Practice (2019) have several guidelines relating to the inclusion of spiritual care in holistic nursing practice, so nurses need to include spiritual care for their holistic nursing care to be aligned with these standards. Therefore, educators could consider the various constructions of spirituality in their spirituality teachings. Additionally, students could be provided the opportunity to reflect on their own spiritual perspectives during education on spirituality. This would be beneficial in helping RNs shape their understanding of spirituality in the context of holistic nursing and enable them to observe and address their patient's spiritual needs without being influenced by their own spiritual perspectives (Mitchell et al., 2006).

In relation to policy, the findings of this study have implications for spirituality's inclusion in policies such as the RN Standards for Practice which governs nursing practice. The RN Standards for Practice serve to provide a structure for registered nursing practice so care provided aligns with these standards (NMBA, 2016). However, including spirituality in practice standards may be difficult with lack of clarity surrounding spirituality's meaning making the provision of spiritual care difficult to measure. Therefore, the question arises regarding whether spirituality should be specifically mentioned in future iterations of the standards or remain as it currently is as an underpinning concept encompassed by related terms such as holistic care and patient-centered care.

### Limitations and Future Research

Considering the sample sizes of both groups of participants, especially the small number of members of the RN Standards for Practice research and development team who participated in this study, the findings may not reflect the perspectives of all RNs. With a larger number of RN participants working within the faith-based private hospital and having a Christian religious background, the findings may reflect more of a Christian worldview. Additionally, this study took place during the COVID 19 pandemic during which nurses may have become more spiritually aware of the sacred space they were working in and drawn more from their personal spirituality to cope with the challenges associated with the pandemic.

Future studies are needed which investigate the resonance between how spirituality is represented in practice standards internationally and RNs constructions of spirituality and experiences in practicing spiritual care.

## Conclusion

The findings of this study revealed there was only one spirituality discourse in common with both groups of participants. This was the discourse of holistic care. The other discourses: person-centered care and professionalization of nursing (members of the RN Standards for Practice research and development team participants) and personal religious beliefs and empathetic care (RNs from both hospitals) were unique to each participant group. The differences in discourses among both participant groups have likely arisen from different power mechanisms. The members of the RN Standards for Practice research and development team participants largely drew from commonly recognized discourses of holistic and person-centered care in health. Whereas the RN participants from both hospitals mostly drew from their religious beliefs, what they were taught in their undergraduate education and their clinical work context. Despite drawing from mostly dissimilar spirituality discourses to the members of the RN Standards for Practice research and development team, the RN participants resonated with the discourse of holistic care. The major findings of this study make a contribution to nursing as they provide a way of understanding how spirituality is constructed in the RN Standards for Practice and how RNs bring meaning to spirituality and spiritual care which has implications for spiritual care education, policy, and practice.
